# Esterified Soy Proteins with Enhanced Antibacterial Properties for the Stabilization of Nano-Emulsions under Acidic Conditions

**DOI:** 10.3390/molecules28073078

**Published:** 2023-03-30

**Authors:** Tingyu Wang, Kehan Yi, Yang Li, Huan Wang, Zhijun Fan, Hua Jin, Jing Xu

**Affiliations:** 1 College of Arts and Sciences, Northeast Agricultural University, Harbin 150030, China; 15245972972@163.com; 2 National Research Center of Soybean Engineering and Technology, Harbin 150028, China; yikehan@neau.edu.cn (K.Y.);; 3 College of Food Science, Northeast Agricultural University, Harbin 150030, China; whname@neau.edu.cn; 4 Heilongjiang Beidahuang Green and Healthy Food Co., Ltd., Jiamusi 154007, China; 15845177666@139.com

**Keywords:** esterification, soy protein, nano-emulsion, acidic, antibacterial

## Abstract

Soy protein isolate (SPI), including β-conglycinin (7S) and glycinin (11S), generally have low solubility under weakly acidic conditions due to the pH closed to their isoelectric points (pIs), which has limited their application in acidic emulsions. Changing protein pI through modification by esterification could be a feasible way to solve this problem. This study aimed to obtain stable nano-emulsion with antibacterial properties under weakly acidic conditions by changing the pI of soy protein emulsifiers. Herein, the esterified soy protein isolate (MSPI), esterified β-conglycinin (M7S), and esterified glycinin (M11S) proteins were prepared. Then, pI, turbidimetric titration, Fourier transform infrared (FTIR) spectra, intrinsic fluorescence spectra, and emulsifying capacity of esterified protein were discussed. The droplet size, the ζ-potential, the stability, and the antibacterial properties of the esterified protein nano-emulsion were analyzed. The results revealed that the esterified proteins MSPI, M7S, and M11S had pIs, which were measured by ζ-potentials, as pH 10.4, 10.3, and 9.0, respectively, as compared to native proteins. All esterified-protein nano-emulsion samples showed a small mean particle size and good stability under weakly acidic conditions (pH 5.0), which was near the original pI of the soy protein. Moreover, the antibacterial experiments showed that the esterified protein-based nano-emulsion had an inhibitory effect on bacteria at pH 5.0.

## 1. Introduction

Generally, nano-emulsions are defined as colloidal systems with a particle size in the range of 50–500 nm that exhibit greater resistance to creaming, sedimentation, coalescence, and flocculation than conventional emulsions, thus exhibiting higher stability [1]. When emulsions are used, they often involve acidic environments (pH 4.0–7.0) [2]. Therefore, whether the emulsion is stable at an acidic pH is a matter of concern. In particular, the improvement of nano-emulsion applications is closely related to the utilization of emulsifiers. As a kind of natural emulsifier with a wide range of sources and select functional properties, soybean protein has been widely used in the preparation of emulsion—based systems [3,4]. However, the solubility and emulsifying properties of soy protein in weakly acidic environments has typically been low, because when the pH value is close to the isoelectric point (pI) of the protein (pI ≈ 4.5), the charge of the protein molecule is almost zero, and the intermolecular repulsion is weak [5], which is not conducive to the application of nano-emulsions made of soybean protein.

Until recently, changing the protein pI by chemical modification has been considered an effective method to improve the solubility of protein near the original pI [6,7,8]. The esterification reaction has blocked the carboxyl groups of the protein, so that the net positive charge of the protein increases, along with the pI, thereby causing a higher solubility of the protein in a low-pH range. Sitohy et al. [9] reported that the pI of esterified soy protein could be increased from 4.5 to 8.0. This was attributed to the formation of esters between the alcohol and carboxyl groups, reducing the content of negatively charged groups on the protein surface.

In addition, esterification is also a feasible way to ensure that a protein achieves antibacterial activity [9,10]. Generally, a protein is positively charged when pH < pI [11]. Blocking the free carboxyl groups (mainly derived from aspartic acid and glutamic acid) of the protein by alcohol ensures that the protein carries positive charges in a wider pH range. The positive charge surface of a protein was then able to act on the negatively charged cell membrane in order to play a role in inhibiting the growth of bacteria [9,12]. Wang et al. [13] found that lactoferrin, after esterification, carried positive charges and had a direct fungitoxic property against the pathogens of blue mold in apple fruit. Sitohy et al. [9] found that esterified legume proteins had a good inhibitory effect on Gram-positive and Gram-negative bacteria due to the presence of static electricity. In conclusion, the esterified proteins exhibited excellent solubility, emulsification, and antibacterial properties under weakly acidic conditions. An emulsion prepared by esterified proteins as an emulsifier had smaller particle sizes, more uniform internal structures, and stronger stability. Therefore, it should be valuable to apply esterification as a modification to improve not only the functional properties of the protein or the preparation of stable emulsions at low pH values but also the antibacterial properties of proteins. However, little is known about the preparation of esterified protein nano-emulsions and their antibacterial properties, so more research is required to explore this process.

In this study, oil-in-water (O/W)-type nano-emulsions (MSPI-NE, M11S-NE, and M7S-NE) were prepared by ethanol-esterified soybean proteins (MSPI, M11S, and M7S). The structures and properties of the esterified proteins were discussed, and the stability and the antibacterial properties of emulsion were also analyzed under weakly acidic conditions. This work provided theoretical proof that soybean proteins could prepare nano-emulsions under weakly acidic conditions and had the dual functions of emulsifier and antiseptic, broadening the prospects for its application in the food-processing, pharmaceutical, and cosmetic industries.

## 2. Results and Discussion

### 2.1. Protein Isoelectric Point (pI)

**Figure 1:**
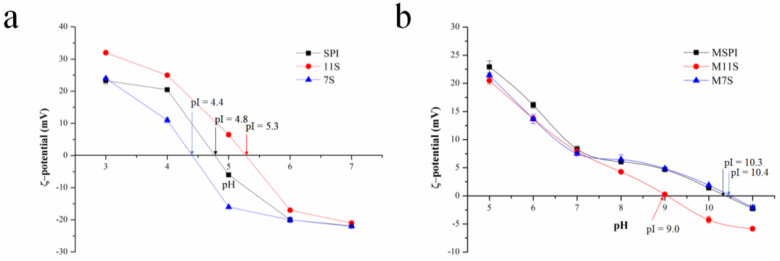
ζ-potetial (mV) of native protein (**a**) and esterified protein (**b**) solution at different pH.

The pI value of protein is often affected by chemical side-chain modifications, the amino-acid composition, and the molecular conformation [14]. The surface charge of the proteins was measured to reveal the pI of the esterified proteins. As shown in Figure 1b, the pI of M11S occurred at a pH of 9.0, according to the zero ζ-potential of M11S at this pH. For the same reason, the pIs of MSPI and M7S occurred at a pH of 10.3 and 10.4, respectively. Therefore, the pI orders of MSPI, M11S, and M7S were as follows: M7S > MSPI > M11S, and the increases were consistent with the orders of the esterification rates. After the reaction between the protein carboxyl groups and ethanol, significantly, the pI of the ethanol-esterified protein had increased due to the negatively charged carboxyl group of the protein being blocked [9]. As shown in Figure 1a, similar results were observed in the esterified soybean, broad-bean, and chickpea protein isolates (pI ≈ 8.0) [9]. Moreover, the lowest pI of M11S could have been related to the compact conformation of 11S linked by an acid such as A polypeptide (37–42 kDa) and a basic B polypeptide (17–20 kDa) via the disulfide bonds [15]. Therefore, it could have had a large number of carboxyl groups embedded in the protein molecule, reducing the number of the carboxyl groups that could react with the ethanol. However, esterification was still an effective method for increasing the positive charges carried by proteins in a wider pH range, especially for SPI and 7S.

### 2.2. Turbidity

**Figure 2:**
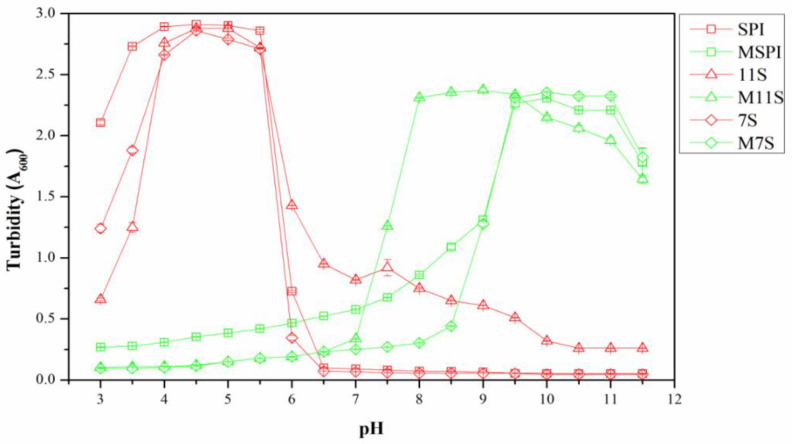
Turbidity curve of native/esterified protein solutions from 3.0 to 11.5 pH.

The turbidity analysis is an intuitive technique to characterize the number and the size of the complex aggregates of proteins [16]. The magnitude of the increase in turbidity depends on the number, the size, and the refractive index of the contrast of the particles [17]. Generally, larger sizes of protein particles dispersed in a solution lead to higher absorbance measurements, which indicate a higher turbidity value. As shown in Figure 2, the turbidity measurements of the native SPI, 11S, and 7S under a range of 4.0–5.5 pH were significantly greater than at other pH values, and this phenomenon revealed the formation of the larger protein particles via pH-induced self-assembly due to their low ζ-potentials and weak electrostatic repulsion [18]. In the range of 3.0-7.0 pH, the turbidity values of the esterified protein solutions were very low, indicating that these were stable solutions. As the pH continued to increase, the turbidity values of all the esterified protein solutions increased significantly, indicating that the esterified proteins did not aggregate at acidic pH values but at alkaline pH values. This verified the results of the pI: The modification by esterification had increased the positive charge of the protein, and thus, the pI had increased.

### 2.3. FTIR Spectra

**Figure 3:**
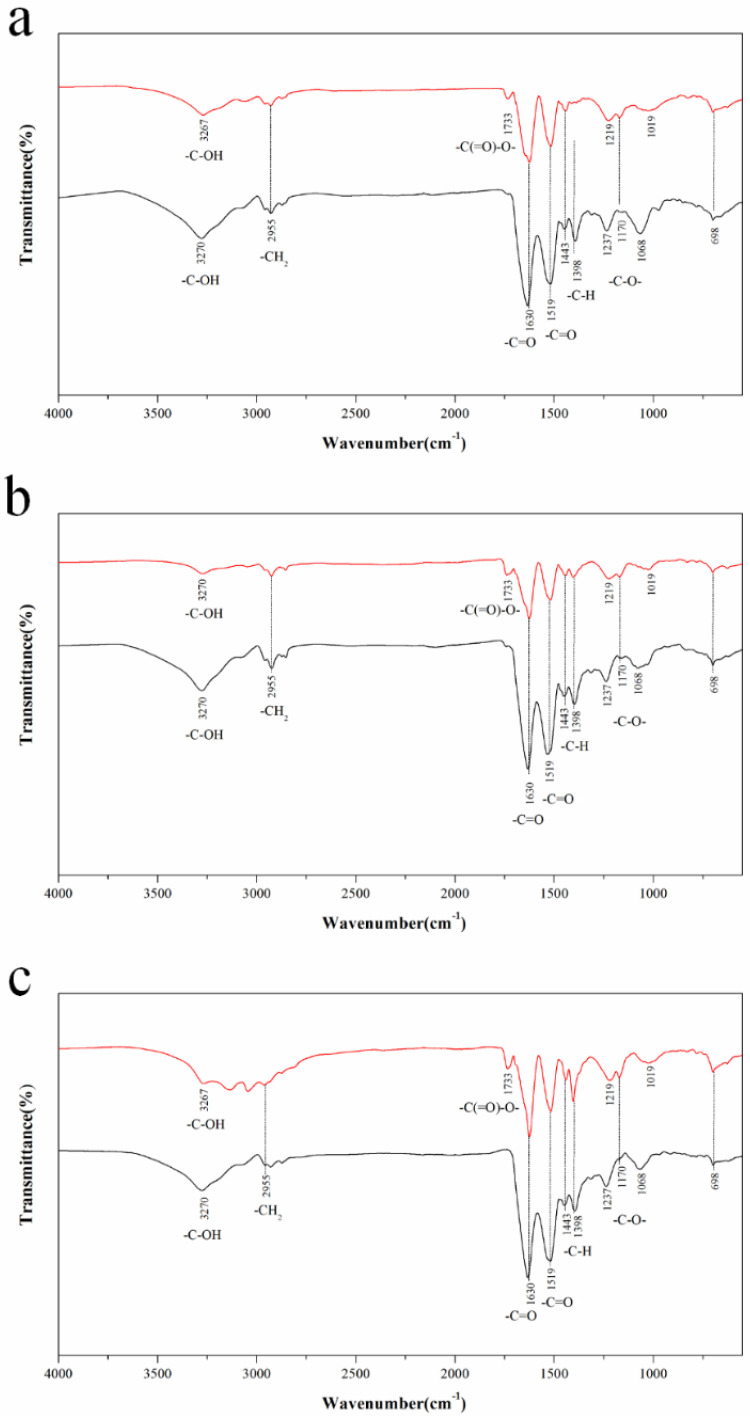
(**a**) FTIR spectra of MSPI (red line) and SPI (black line); (**b**) FTIR spectra of M11S (red line) and 11S (black line); (**c**) FTIR spectra of M7S (red line) and 7S (black line).

To determine the formation of the ester groups in the proteins, the infrared spectrum of the esterified proteins, and the control were measured. The FTIR results of the proteins are shown in Figure 3. A typical broad peak around 3200–3400 cm^−1^ was shown in all the samples due to the intermolecular H–bond and the stretching vibration of O–H and N–H [19]. The absorption peak around 2960 cm^−1^ was attributed to the C–H stretching vibration of the CH_3_ and CH_2_ groups in the protein. The peaks around 1650 and 1540 cm^−1^ in the FTIR spectra of the native proteins were assigned to the C=O stretching vibration and C–N stretching vibration of the amide-I band and the N–H deformation vibration and the stretching vibration of C–N in the amide-II band. At the same time, the esters had 2 characteristically strong absorption bands as a result of the C=O (1750–1735 cm^−1^) and C–O (1300–1000 cm^−1^) stretching [20]. All the esterified proteins (MSPI, M11S, and M7S) showed a small peak at 1733 cm^−1^ due to the C=O absorption band, indicating the formation of the ester groups in the proteins (Figure 3a–c). The results indicated that the esterified proteins were successfully prepared, as these were similar to the results obtained by Wang et al. [20].

### 2.4. Fluorescence Spectra

**Figure 4:**
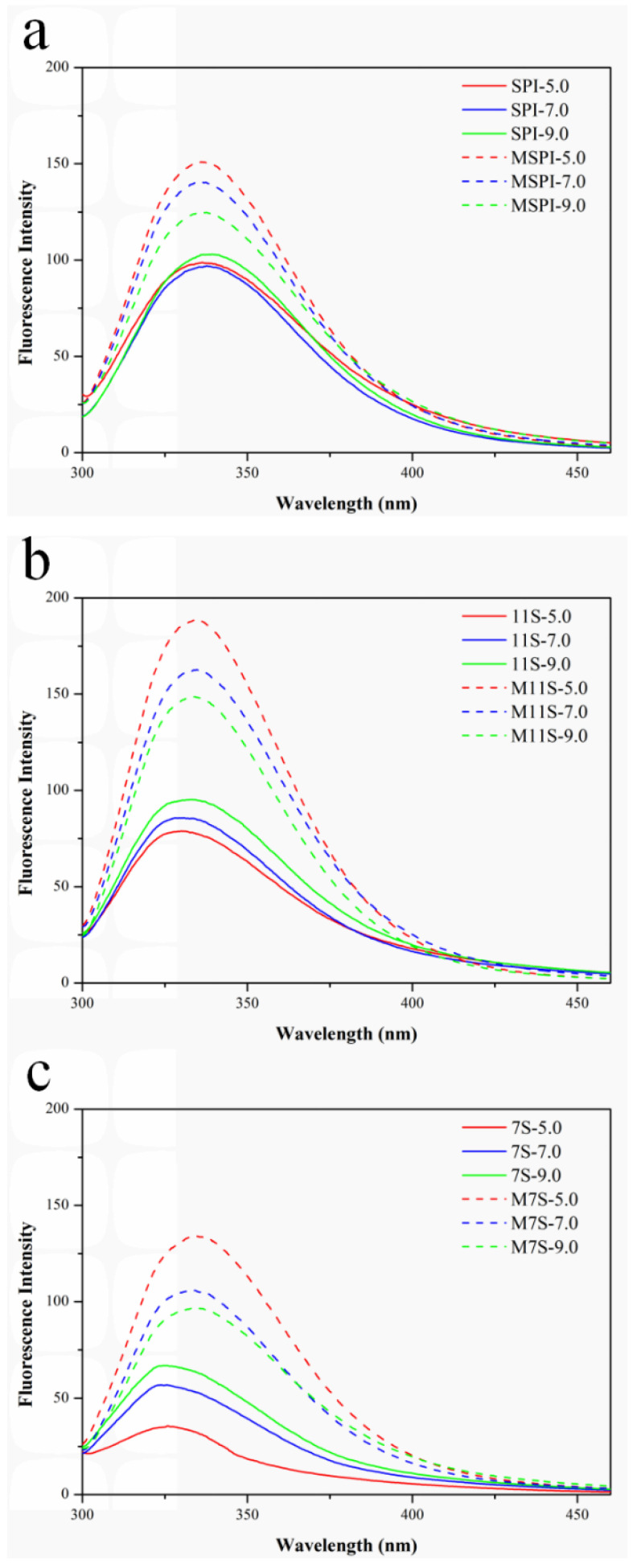
Intrinsic fluorescence at different pH values of MSPI and SPI (**a**); M11S and 11S (**b**); M7S and 7S (**c**).

The structural and polarity information of the proteins was investigated by the intrinsic fluorescence spectra [21]. Generally, the change in the protein conformation in the surrounding environment of the tryptophan residues would be reflected by the changes in the tryptophan fluorescence intensity and the shift trends, depending on whether the tryptophan residues were exposed or buried [22]. As shown in Figure 4a–c, the maximum emission intensity of the protein fluorescence spectrum was greatly affected by the pH. The fluorescence intensity of the native proteins gradually decreased as the pH decreased from 9.0 to 5.0. This phenomenon was attributed to the tryptophan being masked when the native soy proteins aggregated at pH 5.0, as it was then closed to the pI [23]. Similarly, due to the higher pIs of the esterified proteins, MSPI, M11S, and M7S had the lowest fluorescence intensity at pH 9.0. As compared to the native proteins, the higher fluorescence intensity values for the esterified proteins at all the pH values suggested that the tryptophan residues were in the slightly unfolded protein structures of MSPI, M11S, and M7S [23].

### 2.5. Emulsifying Activity Index (EAI) and Emulsion Stability Index (ESI)

**Figure 5:**
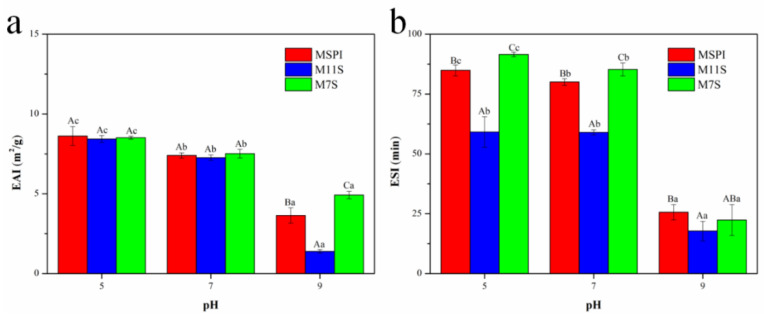
Emulsifying activity index (EAI) (**a**) and emulsion stability index (ESI) (**b**) for esterified soy proteins under different pH conditions. Different capital letters (A–C) indicate that under the same pH conditions, there were significant differences in EAI/ESI results among the different proteins (*p* < 0.05), and different lowercase letters (a–c) indicate that the same protein sample had significant differences in their EAI/ESI values at different pHs (*p* < 0.05).

Native soy protein is a multifunctional protein, which has greatly restricted its application in weakly acidic fields due to its poor water solubility near its pI. In this study, the emulsifying abilities of the esterified proteins were evaluated to analyze the probability of the fabrication of an esterified protein emulsion under weakly acidic conditions. As shown in Figure 5, the emulsifying properties of an esterified protein at 5.0 pH was satisfactory after esterification, which had changed the pI and the solubility, which was similar to the results of Sitohy et al. [10] on esterified β-lactoglobulin. As the pH increased, the EAI values of the esterified soybean protein decreased. This result was attributed to the fewer charges and the worsened solubility of the esterified protein emulsifiers near pI, as the esterified proteins could not fully be absorbed on the surface of the droplets or supply enough electrostatic repulsion between the oil droplets. Until they had reached a pH of 9.0, the emulsifying abilities of the esterified proteins were very low, and the EAI values of MSPI, M11S, and M7S were 3.6, 1.4, and 4.9 m^2^/g, respectively. Moreover, from Figure 5b, we observed that the ESI values of the esterified proteins showed the same trend as EAI. Therefore, the esterification had enhanced the emulsification of soy protein at 5.0 pH, laying a theoretical foundation for the preparation of a stable and uniform emulsion system under weakly acidic conditions. Then, the pHs of 5.0 and 7.0 were selected for subsequent research to further analyze the application potential of the esterified proteins for emulsion preparation.

### 2.6. Physical Properties of Emulsions

#### 2.6.1. Particle Size and ζ-Potential of Emulsions

**Figure 6:**
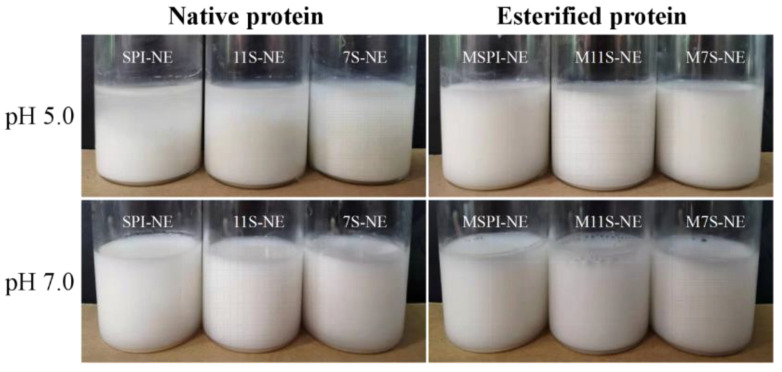
Appearance of oil-in-water emulsions prepared at different pH for native and esterified proteins.

The nano-emulsions were prepared using esterified proteins at pHs of 5.0 and 7.0, and the controls were the emulsions prepared with the corresponding natural proteins. The average particle size and ζ-potential of the different emulsion samples are shown in Table 1. The emulsions prepared from the native proteins were unstable at a pH of 5.0 and separated quickly (Figure 6), with average particle sizes in the micrometer range. However, the emulsions prepared by the esterified proteins were stable and uniformly dispersed at a pH of 5.0 with a small particle size. The average particle diameters of MSPI-NE, M11S-NE, and M7S-NE were 256.9 nm, 438.7 nm, and 257.0 nm, respectively. This difference was consistent with the result of the EAI and ESI. At a pH of 5.0, the ζ-potentials of the emulsions stabilized by MSPI, M11S, and M7S were +26.7, +22.7, and +27.1 mV (Table 1), respectively. They were much higher than the ζ-potentials of +11.2, +6.3, and +11.9 mV, respectively, at a pH of 7.0.The ζ-potentials of the emulsions stabilized by MSPI, M11S, and M7S were +11.2, +6.3, +11.9 mV, respectively, at a pH of 7.0. Therefore, at a pH of 7.0, the mean particle sizes of all the esterified protein nano-emulsions were larger than those at a pH of 5.0. This was attributed by the weakening of the electrostatic repulsion on the surface of the oil droplets, and the oil droplets tended to form larger droplets. There were no significant differences (*p* > 0.05) between the particle sizes of MSPI-NE, as compared to SPI-NE, M7S-NE, and 7S-NE, at a pH of 7.0. This illustrated that the esterified proteins could still stabilize emulsions under neutral conditions because the ζ-potentials of MSPI-NE and M7S-NE were 11.2 and 11.9 mV, respectively, which could still provide a certain amount of electrostatic repulsion. In addition, the surface hydrophobicity of the soy proteins had been reported to increase after modification by esterification [24,25]. As a result, the modification by esterification enabled the soy proteins to be more easily adsorbed at the oil-water interface and form a sturdy interfacial layer, thereby stabilizing the emulsion with a smaller droplet size via strong steric resistance [26].

#### 2.6.2. Emulsion Morphology

**Figure 7:**
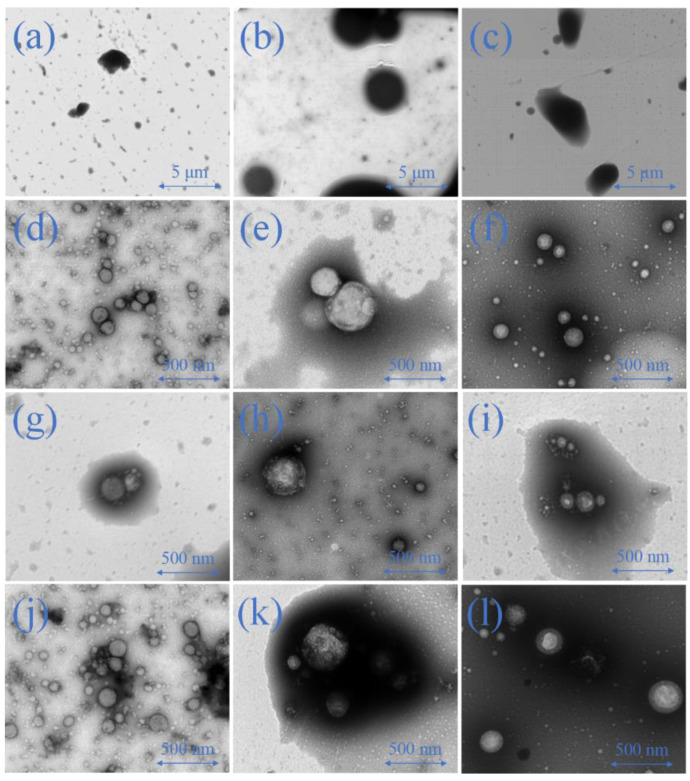
Transmission electron microscopy (TEM) analysis of SPI-NE (pH 5.0, (**a**)), 11S-NE (pH 5.0, (**b**)), 7S-NE (pH 5.0, (**c**)), MSPI-NE (pH 5.0, (**d**)), M11S-NE (pH 5.0, (**e**)), M7S-NE (pH 5.0, (**f**)), SPI-NE (pH 7.0, (**g**)), 11S-NE (pH 7.0, (**h**)), 7S-NE (pH 7.0, (**i**)), MSPI-NE (pH 7.0, (**j**)), M11S-NE (pH 7.0, (**k**)), M7S-NE (pH 7.0, (**l**)).

The microstructures of the esterified protein nano-emulsions were further observed by TEM. First, the images of the microstructures of the native protein emulsions were acquired at a pH of 5.0 (Figure 7a–c). The TEM images showed the flocculation of the oil droplets in the fresh emulsions were stabilized by native proteins, as proteins typically precipitated near the pI [27]. However, the oil droplets of the esterified protein nano-emulsions maintained better spherical microstructures, which was a result of the relatively high electrostatic repulsion and the space resistance between the droplets (Figure 7d–f). At a pH of 7.0, the native protein-based nano-emulsions (Figure 7g–i) and the esterified protein-based nano-emulsions (Figure 7j–l) all displayed regular spherical particles. This suggested that both the esterified and native proteins could be adsorbed on the surface of the oil droplets sufficiently to maintain uniform and stable emulsions. The TEM image results were consistent with the particle-size and ζ-potential results of the emulsions.

### 2.7. Emulsion Stability Analysis

It was important to examine the influence of the storage time and the temperature changes on the stability of the oil-in-water nano-emulsions that had been stabilized by the esterified proteins. This information would be essential for determining the applicable fields for the esterified protein-stabilized nano-emulsions so they could be successfully employed in the food industry.

#### 2.7.1. Storage Stability

**Figure 8:**
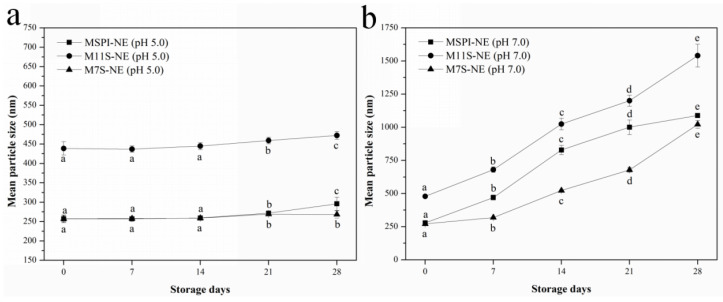
Mean particle sizes (nm) of nano-emulsions during the 28-day storage at 4 °C: pH 5.0 (**a**); pH 7.0 (**b**). Different lowercase letters (a–e) indicate that there are significant differences in the same emulsion after storage (*p* < 0.05).

It is well known that long-term storage can lead to the destabilization of emulsions by flocculation, coalescence, and Ostwald ripening [28], and droplet size was a parameter that assisted in determining the kinetic stability of the emulsions during storage. As shown in Figure 8, at a pH of 5.0, the mean particle sizes had remained relatively small (<500 nm) during the 28-day storage period, although there had been an appreciable increase on the 21st day in the esterified-protein nano-emulsions (Figure 8a). The particle size results indicated that the esterified protein had the ability to maintain stable nano-emulsions under weakly acidic conditions. In addition, considering the highest esterification rates and ζ-potentials, the modification by esterification could have had the strongest effect on the surface charge and the hydrophobicity of 7S. Therefore, M7S showed the strongest ability to generate electrostatic repulsion and supply space resistance; thus, the emulsion prepared by M7S showed the best stability at the end of storage.

Though the esterified proteins had prepared nano-emulsions with small particle sizes, the particle size of the emulsion increased significantly after 7 days at a pH of 7.0 (Figure 8b). There was appreciable droplet aggregation in the emulsions because of the poor electrostatic repulsion induced by the low net-charge of the droplets at a pH of 7.0 (Table 1), which could not, therefore, stabilize the emulsion in long-term storage. The relationship between the emulsion stability and the electrostatic repulsion had been confirmed in many studies. For example, Wang et al. [29] found a similar result: Emulsions stabilized by soy protein isolates complexes showed weak electrostatic repulsions, so the particle size of the emulsion had changed significantly after storage.

#### 2.7.2. Temperature Treatment

**Figure 9:**
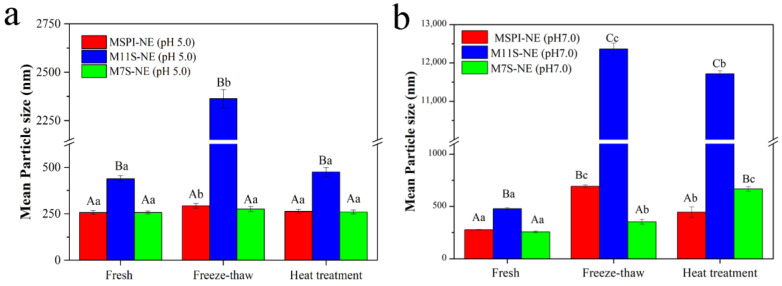
Mean particle size of fresh, freeze-thawed, or heat-treated nano-emulsions at a pH of 5.0 (**a**) and a pH of 7.0 (**b**); Different capital letters (A–C) indicate significant differences in mean particle size between different nano-emulsions under the same treatment (*p* < 0.05), and different lowercase letters (a–c) indicate significant differences (*p* < 0.05) in the mean particle size of the same emulsion sample under different treatments.

Emulsion products may be subjected to various effects of temperature fluctuations during industrial production, such as high-temperature sterilization or low-temperature storage to extend their shelf lives. Therefore, it was important to determine the influence of temperature on the particle size of the esterified protein nano-emulsions. For the soy proteins, the temperature conditions could lead to conformational changes, exposing the interior hydrophobic patches and decreasing solubility [30,31]. The particle sizes of the emulsions after high-temperature heating and freeze-thaw treatments were measured, as shown in Figure 9. After freeze-thaw treatment, all nano-emulsions produced oil droplets with larger particle sizes by coalescence and flocculation, though to a different extent (Figure 9a,b). At a pH of 5.0, the mean particle sizes of MSPI-NE and M7S-NE increased from 256.9 nm to 293.0 nm and from 257.0 nm to 275.8 nm, respectively. Accordingly, the small increments in mean particle sizes suggested that MSPI-NE and M7S-NE had acceptable freeze-thaw stability. The phenomenon of droplet aggregation during freezing and thawing was mainly related to the destruction of the emulsifier layer by the ice crystals. The modification by esterification improved the hydrophobicity of the proteins [21], so the adsorption of the proteins at the oil-water interface became strong, contributing to the stability of the nano-emulsions. However, the droplets of M11S-NE gathered significantly (>2 µm), which was attributed to the properties of 11S that precipitates after the freezing treatment [16,32]. The 11S-protein molecules tended to aggregate at low temperatures, which caused the emulsifier layer at the oil-water interface to be insufficient for the stabilization of the nano-emulsions. In addition, during freezing, the concentration of the ions in the unfrozen water phase could increase, resulting in electrostatic shielding that promoted a decrease in the electrostatic repulsion between the droplets [33]. This could explain why the particle sizes of the nano-emulsions with a pH of 7.0 increased more significantly. Because of the relatively low net-charge of the droplets at a pH of 7.0, they were more affected by the electrostatic shielding.

After being treated at 95 °C for 30 min, the mean particle sizes of MSPI-NE, M11S-NE, and M7S-NE changed from 256.9 nm, 438.7 nm, and 257.0 nm, respectively, to 263.6 nm, 475.2 nm, and 260.1 nm (Figure 9a), respectively, indicating that the effect of the heat treatment had been limited at a pH of 5.0. On the one hand, the modification by esterification improved the emulsification of the native proteins under acidic conditions, the proteins could more easily be adsorbed on the surface of the oil droplets to form compact interface layers in order to stabilize the emulsion through steric hindrance [10]. On the other hand, the charge densities of the esterified proteins were higher at a pH of 5.0, which provided sufficient electrostatic repulsion to inhibit the aggregation of the emulsion droplets. In addition, the tertiary structures of the proteins unfolded after proper heat treatments, and then the flexibility and the emulsification of the protein molecules increased, which was also beneficial for the emulsion stability [34]. Regarding the thermal stability, M11S-NE still showed the worst results among all the esterified protein nano-emulsions. According to the above experimental results, the lower positive charges on the droplet surfaces of M11S-NE (Table 1) may not have been enough to inhibit the aggregation of the droplets after the heat treatment. In summary, the three esterified proteins were effective in stable emulsions, but the modification of the densely structured 11S showed a slightly lower nano-emulsion stability than SPI and 7S.

### 2.8. Bacteriostatic Analysis of Nano-Emulsion

In recent years, many studies have confirmed that esterified proteins have the functions of inhibiting and killing bacteria and microorganisms due to their positive charges, which act on the negatively charged cell membrane to restrain the growth of bacteria [9,13,35]. In this experiment, the esterified soy proteins appeared to have dual functions as emulsifiers and bacteriostatic agents.

**Figure 10:**
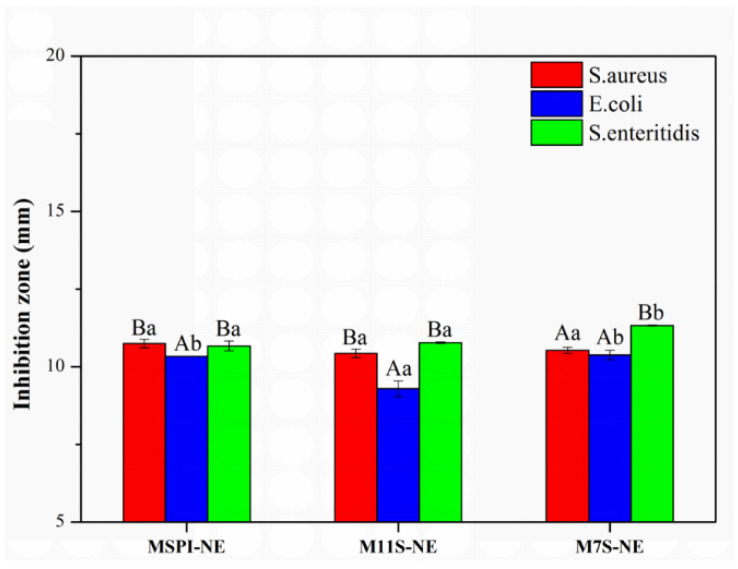
Diameters (mm) of the antibacterial zone formed by nano-emulsions at a pH of 5.0. For the same bacteria, values of different nano-emulsions followed by different lowercase letters mean significant differences (*p* < 0.05); for the same nano-emulsion sample, values of different bacteria followed by different capital letters mean significant differences (*p* < 0.05).

It was found that the esterified protein-based nano-emulsions had an inhibitory effect on the 3 kinds of bacteria, as shown in Figure 10, but no antibacterial activity was found in any of the control samples. This result was attributed to the fact that the esterified protein carried a positively charged amino group and the bacterial surface potential was usually negative [9]. The positively charged droplets could interact with the cell membrane electrostatically, and then, the life activities of the bacteria were disturbed. As compared to the microbial inhibition zone (Figure 10), we found that M7S-NE and MSPI-NE had a good inhibitory effect on *S*. *aureus* and *S*. *enteritidis*. The diameters of the inhibitory zone of MSPI-NE were 10.74 mm and 10.66 mm, and the diameters of the inhibitory zone of M7S-NE were 10.55 mm and 11.32 mm. The inhibitory effect of the acid emulsion on *E*. *coli* was lower, and the inhibitory zone of MSPI-NE, M11S-NE, and M7S-NE were 10.33 mm, 9.29 mm, and 10.38 mm, respectively. This result may have been related to the good growth trend of *E. coli*. M11S-NE had the lowest amount of positive charge, and it also had a degree of antibacterial activity against *E. coli*. Generally, the more positive the charge carried by the bacteriostatic agent, the stronger the electrostatic interaction with the cell membrane, and the easier it was to adsorb around the bacteria and destroy the morphology of the cell membrane [36,37]. Sitohy et al. [9] found that an esterified legume-protein solution had strong antibacterial properties due to the presence of a large amount of positive charges that could act on the cell membrane. However, for proteins with low rates of esterification, such as an esterified broad-bean protein (MBPI), the small electrostatic force between the protein and the cell membrane led to the weaker antibacterial activity of MBPI.

In addition, the antibacterial properties of MSPI-NE, M11S-NE, and M7S-NE were undiscovered at a pH of 7.0. As shown in Table 1, the positive charges of the nano-emulsions were high (MSPI-NE: 26.7 mV; M11S-NE: 22.7 mV; M7S-NE: 27.1 mV) at a pH of 5.0, but they were significantly decreased at a pH of 7.0 (MSPI-NE: 11.2 mV; M11S-NE: 6.3 mV; M7S-NE: 11.9 mV), which could have reduced the electrostatic interaction at a pH of 7.0 between the esterified proteins and the bacteria [38]. In summary, the esterified proteins had higher positive charged under acidic conditions, and therefore, they could inhibit the growth of bacteria, but the bacteriostasis of the proteins was weakened or even lost as the pH increased.

In the future, we will study the delivery characteristics of the esterified protein nano-emulsions further. The esterified protein nano-emulsions could be embedded with bioactive substances. And established an in vitro digestion model to investigate the digestion characteristics of the esterified protein nano-emulsions, which will be investigated at three digestion stages, including at the mouth, in the stomach, and in the small intestine.

## 3. Materials and Method

### 3.1. Materials

Low-temperature defatted soybean meal was bought from the Zhaoyuan Food Factory, Shandong, China. *Staphyloccocus aureus* (*S. aureus*), *Escherichia coli* (*E. coli*), and *Salmonella enteritidis* (*S. enteritidis*) were obtained from the China General Microbiological Culture Collection Center. Corn oil was purchased from the Yihai Kerry foodstuffs marketing company (Harbin, China). All chemicals used in this study were of analytical grade.

These were all the samples used in this study: soybean protein isolate (SPI), β-conglycinin (7S), glycinin (11S), esterified soybean protein isolate (MSPI), esterified β-conglycinin (M7S), esterified glycinin (M11S), nano-emulsion prepared by esterified soy protein isolate (MSPI-NE), nano-emulsion prepared by esterified β-conglycinin (M7S-NE), and nano-emulsion prepared by esterified glycinin (M11S-NE).

### 3.2. Protein Sample Preparation

The soy protein isolate (SPI), β-conglycinin (7S), and glycinin (11S) proteins were extracted according to previous research methods [39,40]. The precipitate was washed 3 times, dissolved with ultrapure water to pH 7.0, and then dialyzed for 3 days and lyophilized. The protein contents of SPI, 7S, and 11S were determined by the Kjeldahl method [12] (N × 6.25) to be 91.53 ± 1.27%, 91.74 ± 1.58%, and 93.62 ± 1.35% *w*/*w*, respectively.

### 3.3. Protein Esterification

**Figure 11:**
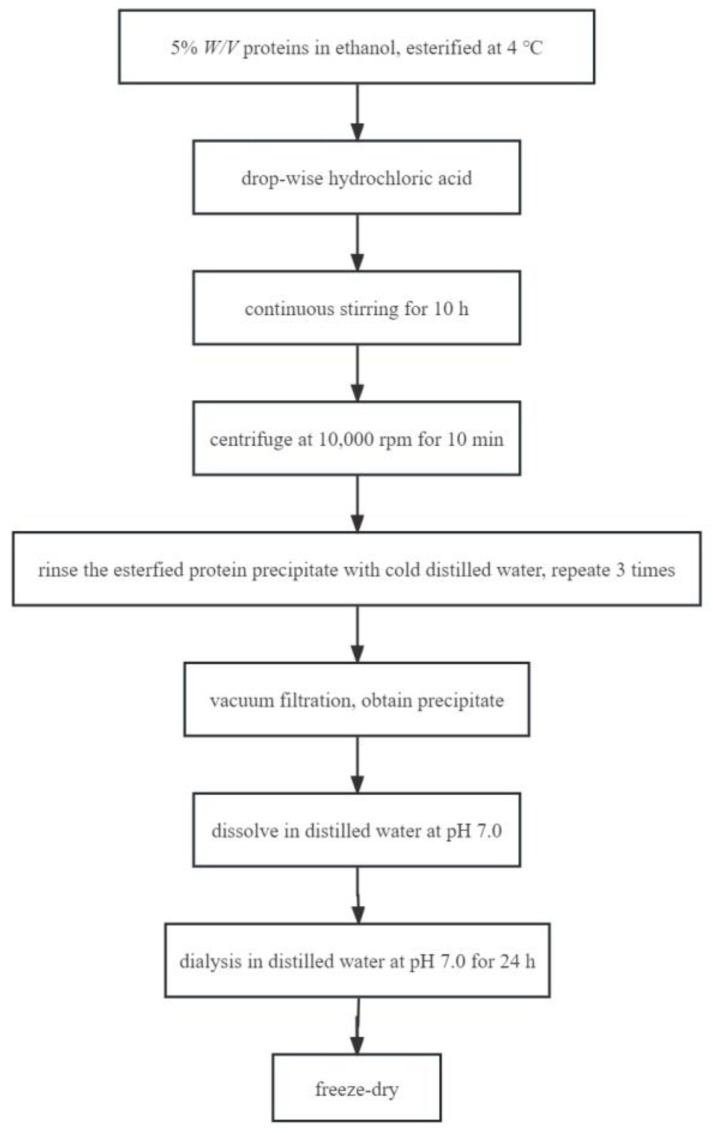
Flowchart of preparation of esterified protein.

Samples were prepared following the procedure of Sitohy et al. [9], with some modifications. Esterified proteins preparation is shown in Figure 11. All proteins (SPI, 7S, or 11S) were esterified at 4 °C by dispersing 5% *w*/*v* proteins in ethanol (>99.5%). The amount of aspartic acid (Asp) and glutamic acid (Glu) contents in SPI, 11S, and 7S samples were determined by amino acid analysis to calculate the amount of free carboxyl [41]. Amounts of hydrochloric acid equivalent to 50 molar ratio (acid H^+^/COO^−^) were added drop-wise at the beginning of the reaction to induce the protonation of the carboxylate on protein. After continuous stirring for 10 h, the samples were centrifuged at 10,000 r/min for 10 min. The esterified protein precipitate was dispersed in cold distilled water and rinsed repeatedly three times to remove residual ethanol and hydrochloric acid. Vacuum filtration was used instead of centrifugation to obtain precipitate, and then the precipitate was dissolved in distilled water at pH 7.0. Finally, the samples were dialyzed against distilled water at 4 °C for 24 h and then lyophilized. The amount of carboxyl groups that had been esterified by ethanol were determined based on the formation of colored hydroxymate-ferric ion chelate [2]. Modification was quantified based on the standard curve for the molar adsorption of iron chelate (R^2^ = 0.99) that was prepared using ethyl thioacetate as a standard. In this study, the esterification rate of esterified proteins MSPI, M11S, M7S was 75.49 ± 0.36%, 69.64 ± 0.43%, and 78.16 ± 0.25%, respectively.

### 3.4. Isoelectric Point (PI) Determination of Esterified Proteins

The method of Wang et al. [7] was referenced and slightly modified. The native and esterified protein samples were dissolved in deionized water, and the ζ-potentials of different protein dispersions (1 mg/mL) at pH 3–11 were determined using a Zetasizer Nano ZS90 instrument (Malvern Instruments Ltd., Worcestershire, UK). A total of 0.1, 0.5, and 1 M hydrochloric acid (HCl) solutions or sodium hydroxide (NaOH) solutions were used for pH adjustment. The pI was considered to be the pH corresponding to a zero ζ-potential in the ζ-potential-versus-pH curve.

### 3.5. Turbidimetric Titration

The method of Dong et al. [42] was referenced and slightly modified. The pH values of native and esterified proteins (1 mg/mL) were adjusted from 3.0 to 11.5, varying by 0.5 units, and the turbidity was measured at each pH to form a turbidity titration curve. Turbidity was expressed as the absorbances of the samples at 600 nm, which were measured using a UV-2450 spectrophotometer (Shimadzu, Kyoto, Japan).

### 3.6. Structural Analysis of Proteins

#### 3.6.1. Fourier Transform Infrared (FTIR) Analysis

The samples were analyzed by FTIR spectrometer (IRTracer-100, Shimadzu, Kyoto, Japan), operating at 0.09 cm^−1^ resolution with 64 scans per test. Native and esterified soy protein samples (2 mg) were mixed with kalium bromatum (KBr) (200 mg) and placed under the probe, and spectroscopic data were collected from 4000 to 400 cm^−1^ in the absorbance mode [43].

#### 3.6.2. Intrinsic Fluorescence Spectra

The intrinsic fluorescence spectra of native and esterified protein solutions (0.5 mg/mL) at pHs of 5.0, 7.0, and 9.0 were acquired using an F-4700 fluorescence spectrophotometer (Hitachi, Tokyo, Japan). The protein was excited at 290 nm, and the fluorescence intensity in the wavelength range of 300–500 nm was recorded as the emission spectrum. Both the excitation slit and the emission slit were 5 nm [44].

### 3.7. Emulsifying Capacity of Proteins

Emulsion stability (ESI) and activity indexes (EAI) were measured according to the modified procedure of Ellouze and Pearce et al. [45,46]. Esterified protein solution (1%, *w*/*v*) adjusted to pHs of 5.0, 7.0, and 9.0 was mixed with corn oil at a ratio of 3:1 (*v*/*v*) and homogenized with a high-speed homogenizer (UltraTurrax T25, IKA, Staufen, Germany) at 10,000 r/min for 2 min. After standing for 0 and 10 min, 40 μL aliquots of the emulsion were transferred to 10 mL of 0.1% (*w*/*w*) sodium dodecyl sulfate (SDS). Optical density was recorded at 500 nm using a UV-2450 spectrophotometer (Shimadzu, Kyoto, Japan). EAI and ESI were then calculated using Equations (1) and (2), respectively:

(1) $${\mathrm{EAI}\;(m}^{2}/g)=\frac{2\;\times \;2{.303\;\times \;A}_{1}\;\times \;N}{c\;\times \;(1\;-\;\varphi )\;\times \;10000}$$

(2) $$\mathrm{ESI}\;(\min)=\frac{A_{1}}{A_{1}-{\;A}_{2}}\times 10$$

where *A*_1_ is the absorbance of the diluted emulsion immediately after homogenization, *N* is the dilution factor (250), *c* is the weight of protein per volume (g/mL), *φ* is the oil volume fraction in the emulsion (25%), and *A*_1_ *− A*_2_ is the difference of the absorbance between time 0 and time 10 min.

### 3.8. Emulsion Properties

#### 3.8.1. Preparation

Protein solutions (1%, *w*/*v*) at pHs of 5.0 and 7.0 were used as the water phase, and corn oil was used as the oil phase. The oil and water phase were mixed at a ratio of 2:98 (*v*/*v*), homogenized at 10,000 r/min for 4 min, and then sonicated by ultrasound probe for 20 min at 500 W using Scientz-II D ultrasound generator (Scientz Biotechnology Co., Ltd., Ningbo, China). During sonication, the temperature of the samples was maintained below 20 °C by utilizing an ice-water bath.

#### 3.8.2. Potential and Particle Size Measurements

The ζ-potentials and droplet sizes of the nano-emulsions with different pHs were determined by a Zetasizer nano-zs90 instrument (Malvern Instruments Ltd., Worcestershire, UK) and a Mastersizer 2000 instrument (Malvern Instruments Ltd., Worcestershire, UK), respectively. The parameter settings of the refractive indices were oil phase (1.46) and water phase (1.33). Samples were diluted 100-fold with deionized water to avoid multiple scattering effects and used for analysis [47].

#### 3.8.3. Microstructure Observation

The microstructure of the emulsions was evaluated by transmission electron microscopy (TEM) (EM 902A, ZEISS, Oberkochen, Germany). The nano-emulsion was diluted to a certain multiple. Then, the diluted nano-emulsion sample was dropped onto a 200-meshed carbon-coated copper grid. The nano-emulsion was stained with 1% (*w*/*v*) phosphotungstic acid and dried at room temperature for 10 min before being observed using an accelerating voltage of 100 kV.

### 3.9. Stability of Emulsion

The stability of emulsion was observed by measuring the particle size of emulsion. First, we studied the stability of emulsion under different pH conditions; second, we studied the stability of the emulsion after heat treatment (95 °C, 30 min); third, we studied the stability of the emulsion after freeze-thaw treatment (−20 °C, 24 h); and finally, we studied the storage stability of emulsion (4 °C, 28 days). Please refer to Section 3.8.2 for the determination of emulsion particle size.

### 3.10. Agar Well Diffusion

According to the modified method of Yuan et al. [48], we evaluated the antibacterial effect of esterified protein emulsion. The 100 mL of inoculum (10^6^ CFU/mL) was uniformly spread across the poured nutrient agar medium. Oxford cups of 7.8 mm diameter were filled with 150 µL of the test emulsion and left at 4 °C for 12 h. The petri dish was incubated for 24 h at 36 ± 1 °C. Additionally, the emulsions containing the same concentration of native protein (pH 5.0 and 7.0) were prepared as a control. The zone diameter was measured by a caliper.

### 3.11. Statistical Analysis

All experiments were performed in triplicate and all data were presented as mean ± standard deviation (SD). Data were subjected to one-way ANOVA and Duncan’s significant difference analysis using SPSS software (IBM SPSS statistic 19). The significant level (*p*) was set as 0.05.

## 4. Conclusions

In conclusion, the modification by esterification of soy proteins with ethanol changed the structures of the soy proteins, increased the isoelectric point of the soy proteins, and effectively promoted the emulsification of the soy proteins at a low pH range. Under weakly acidic conditions, the esterified soy protein nano-emulsions exhibited excellent storage stability, freeze-thaw stability, and thermal stability, as compared to natural soy protein nano-emulsions. In addition, the modification by esterification also resulted in antibacterial properties in the esterified protein nano-emulsions at a pH of 5.0. Therefore, esterification could broaden the application of the proteins in emulsion delivery systems under low pH conditions.

## Figures and Tables

**Table 1:** Mean particle sizes (nm) and ζ-potentials (mV) of oil-in-water nano-emulsions stabilized by various proteins at different pH values.

Sample	Mean Particle Size (nm)		ζ-Potential (mV)	
	pH 5.0	pH 7.0	pH 5.0	pH 7.0
SPI-NE	3500 ± 100 ^c^	278.2 ± 5.5 ^a^	−1.7 ± 0.1 ^b^	−33.2 ± 1.8 ^a^
MSPI-NE	256.9 ± 10.1 ^a^	278.4 ± 3.0 ^a^	26.7 ± 0.5 ^e^	11.2 ± 0.3 ^d^
11S-NE	5130 ± 170 ^e^	357.8 ± 8.2 ^b^	1.2 ± 0.1 ^c^	−30.6 ± 0.5 ^b^
M11S-NE	438.7 ± 17.2 ^b^	479.0 ± 9.7 ^c^	22.7 ± 0.2 ^d^	6.3 ± 0.1 ^c^
7S-NE	3840 ± 140 ^d^	273.6 ± 8.4 ^a^	−1.4 ± 0.1 ^a^	−32.6 ± 0.7 ^a^
M7S-NE	257.0 ± 8.1 ^a^	270.8 ± 7.5 ^a^	27.1 ± 0.1 ^e^	11.9 ± 0.6 ^d^

Data not sharing the same superscript letter in the same column are significantly different (*p* < 0.05).

## Data Availability

Not applicable.
